# Identification of Cognitive Superaging and its Predictors Among Older Adults: A Latent Profile Analysis

**DOI:** 10.1093/geroni/igaf122.3607

**Published:** 2025-12-31

**Authors:** Maham Khan, Laura Sands, Benjamin Katz

**Affiliations:** Virginia Tech., Blacksburg, Virginia, United States; Virginia Tech, Blacksburg, Virginia, United States; Virginia Tech, Blacksburg, Virginia, United States

## Abstract

Older adults demonstrate substantial variability in cognitive functioning, with some maintaining exceptional performance, called Cognitive SuperAging (CSA). This study identified cognitive superagers and other cognitive profiles among individuals ≥ 75 years in 2020 Health and Retirement Study, and examined demographic, health, and behavioral predictors of CSA. The sample included 5,646 individuals with scores on different cognitive domains, including memory, attention, executive functioning (EF), and fluid intelligence-related tasks. Latent Profile Analysis models with 3-6 profiles were compared using fit indices (AIC, BIC, adjusted-BIC, entropy). A four-profile solution best fit the data, comprising: Class 1 Superagers (11.5%), Class 2 Average Memory, Impaired Attention & EF (39.9%), Class 3 Impaired Memory & EF, High Attention (37.2%), and Class 4 Globally Impaired (11.4%). A conditional model using R3STEP indicated sharpest separation between Globally Impaired and Superagers (Entropy = 0.77). The likelihood of not being Superager increased by 27% with age, 15% with each additional chronic condition, and nearly doubled with higher depressive symptoms. Higher physical activity was associated with more than twice the odds of being in Superaging profile (OR = 2.43, 95%CI [2.25, 2.64]). Biological sex showed no significant association, and sleep disturbance was associated with marginal negative effects (OR = 1.033, 95%CI [1.005 – 1.063]) on superaging. The intermediate profiles also showed similar but less pronounced trends. The results have implications for interventions and policies aimed at promoting resilience in cognitive aging. Findings support targeting physical activity, mental health, and chronic disease management to help maintain high cognitive functioning into advanced age.

